# Smart Clothing and Medical Imaging Innovations for Real-Time Monitoring and Early Detection of Stroke: Bridging Technology and Patient Care

**DOI:** 10.3390/diagnostics15151970

**Published:** 2025-08-06

**Authors:** David Sipos, Kata Vészi, Bence Bogár, Dániel Pető, Gábor Füredi, József Betlehem, Attila András Pandur

**Affiliations:** 1 Department of Medical Imaging, Faculty of Health Sciences, University of Pécs, 7400 Kaposvár, Hungary; 2 Dr. József Baka Diagnostic, Radiation Oncology, Research and Teaching Center, “Moritz Kaposi” Teaching Hospital, Guba Sándor Street 40, 7400 Kaposvár, Hungary; 3 Department of Oxyology and Emergency Care, Pedagogy of Health and Nursing Sciences, Institute of Emergency Care, Faculty of Health Sciences, University of Pécs, 7624 Pécs, Hungaryattila.pandur@etk.pte.hu (A.A.P.)

**Keywords:** stroke, smart clothing, real-time monitoring, early detection, medical imaging

## Abstract

Stroke is a significant global health concern characterized by the abrupt disruption of cerebral blood flow, leading to neurological impairment. Accurate and timely diagnosis—enabled by imaging modalities such as computed tomography (CT) and magnetic resonance imaging (MRI)—is essential for differentiating stroke types and initiating interventions like thrombolysis, thrombectomy, or surgical management. In parallel, recent advancements in wearable technology, particularly smart clothing, offer new opportunities for stroke prevention, real-time monitoring, and rehabilitation. These garments integrate various sensors, including electrocardiogram (ECG) electrodes, electroencephalography (EEG) caps, electromyography (EMG) sensors, and motion or pressure sensors, to continuously track physiological and functional parameters. For example, ECG shirts monitor cardiac rhythm to detect atrial fibrillation, smart socks assess gait asymmetry for early mobility decline, and EEG caps provide data on neurocognitive recovery during rehabilitation. These technologies support personalized care across the stroke continuum, from early risk detection and acute event monitoring to long-term recovery. Integration with AI-driven analytics further enhances diagnostic accuracy and therapy optimization. This narrative review explores the application of smart clothing in conjunction with traditional imaging to improve stroke management and patient outcomes through a more proactive, connected, and patient-centered approach.

## 1. Introduction

Stroke is a leading cause of disability and mortality worldwide, characterized by the abrupt disruption of blood flow to the brain, resulting in neurological damage. This medical emergency demands precise diagnosis and timely intervention to minimize the extent of brain injury and improve patient outcomes [1]. In 2023, the global burden of stroke continued to rise, underscoring the urgent need for innovative prevention and care strategies. Strokes are broadly classified into ischemic and hemorrhagic types, each with distinct etiologies and treatment approaches. Ischemic strokes, accounting for the majority of cases, result from blood vessel occlusion, while hemorrhagic strokes stem from vascular rupture [2]. Early recognition of symptoms, such as weakness, aphasia, or sudden headache, alongside state-of-the-art advanced imaging techniques, is critical in determining the type and guiding treatment [1,2,3].

Innovations in healthcare technology, particularly wearable devices and smart clothing, are revolutionizing stroke care [4,5]. Recent advancements in 2024 include devices with enhanced AI integration and improved sensor accuracy, enabling continuous real-time monitoring. These advancements are enhancing patient outcomes through real-time monitoring, data-driven rehabilitation, and personalized therapy. Examples include smart garments embedded with sensors to track vitals, motion, and neural activity, offering invaluable support for early detection, therapy adjustments, and long-term recovery [6,7,8]. Additionally, emerging technologies like portable MRI scanners and AI-assisted stroke detection software (Brainomix [5]) are transforming acute stroke management, bridging gaps in accessibility for underserved regions.

The aim of this narrative review is to explore the integration of traditional stroke diagnostics and advanced medical imaging with emerging wearable technologies, such as smart clothing, to improve stroke prevention, rehabilitation, and management. This review seeks to provide a comprehensive understanding of how these interdisciplinary innovations enhance patient outcomes through real-time monitoring, data-driven therapeutic adjustments, and personalized care strategies. By bridging the gap between conventional and modern healthcare approaches, the review aims to highlight the potential of these advancements to transform stroke care into a more effective and patient-centered system.

### Methodology

This review was conducted to provide a comprehensive analysis of the integration of wearable technologies and advanced medical imaging in stroke care. A systematic approach was employed to identify relevant literature across multiple databases, including PubMed, Scopus, and Web of Science. The search strategy used a combination of keywords such as “stroke”, “smart clothing”, “wearable technology”, “real-time monitoring”, “medical imaging”, and “rehabilitation”. Articles published in English from 2010 to 2024 were included to capture recent advancements and trends. Inclusion criteria were peer-reviewed studies that focused on the application of wearable technologies or medical imaging in stroke diagnosis, monitoring, prevention, or rehabilitation. Exclusion criteria included articles not directly related to stroke care, studies with insufficient data on clinical outcomes, and non-peer-reviewed sources such as conference abstracts or editorials. The initial search yielded 350 articles, which were screened based on titles and abstracts. Full-text reviews were performed on 120 articles, resulting in the final inclusion of 75 studies that met all criteria. This rigorous methodology ensures a comprehensive and credible analysis of the current state of wearable technologies and advanced imaging in stroke care.

## 2. Overview of Most Common Stroke Types

Stroke is a neurological emergency caused by the sudden disruption of blood flow to the brain, leading to cellular injury and potential loss of neurological function [7]. Broadly, strokes are classified into two main types: ischemic and hemorrhagic. Ischemic stroke, which accounts for approximately 87% of all strokes, occurs due to obstruction of cerebral blood flow, commonly caused by thromboembolism or large vessel atherosclerosis [7,8,9,10]. Hemorrhagic stroke results from ruptured blood vessels leading to intracerebral or subarachnoid bleeding, often associated with hypertension or aneurysmal rupture. Timely identification of stroke type is critical, as management strategies differ significantly for ischemic and hemorrhagic strokes [11,12,13,14,15,16].

### 2.1. Stroke Symptoms and Diagnosis

Stroke symptoms often occur suddenly and require immediate recognition to improve outcomes. Key symptoms include sudden weakness or numbness (particularly on one side of the body), difficulty speaking or understanding speech (aphasia), facial drooping, sudden vision changes, severe headache (often linked to hemorrhagic stroke), and loss of coordination or balance. These symptoms demand rapid response and intervention, as every minute of delay contributes to worsening brain injury. Public awareness initiatives, such as the FAST (Face drooping, Arm weakness, Speech difficulties, Time to call emergency services) protocol, emphasize the importance of prompt action [17,18,19,20].

**Figure 1:**
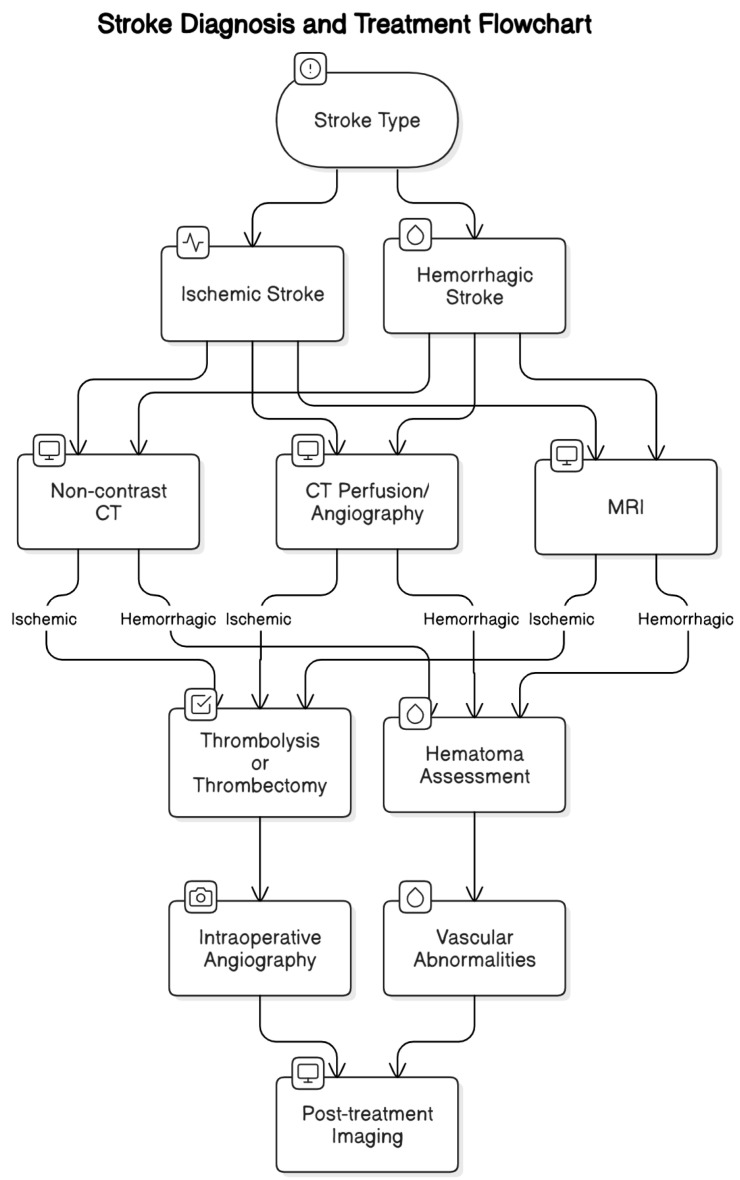
Stroke Diagnosis and Treatment Flowchart.

Diagnosis relies on rapid imaging to differentiate stroke types. Non-contrast computed tomography (CT) is the first-line modality due to its speed and availability, while magnetic resonance imaging (MRI), particularly diffusion-weighted imaging, is highly sensitive for early ischemic changes. These imaging modalities guide acute treatment decisions, underscoring the principle of “time is brain” and the need for swift action in stroke management [21,22,23] (Figure 1).

### 2.2. Imaging Modalities Used in Stroke Diagnosis

Imaging is critical for identifying stroke type, location, and severity. Non-contrast CT distinguishes ischemic strokes (hypoattenuation) from hemorrhagic strokes (hyperdense regions). Advanced techniques like CT angiography and perfusion imaging assess blood flow and vascular integrity, identifying salvageable brain tissue and guiding interventions like thrombectomy [20,22,23,24]. MRI, particularly with diffusion-weighted imaging, is highly sensitive to ischemic changes and offers additional insights into vascular abnormalities without ionizing radiation. Transcranial Doppler ultrasound and MR/CT angiography provide further vascular assessments, supporting timely and accurate treatment decisions [20,25,26,27].

### 2.3. Analysis of Imaging for Treatment

In ischemic stroke, imaging helps determine eligibility for time-sensitive interventions like intravenous thrombolysis with tissue plasminogen activator (tPA) or mechanical thrombectomy [28]. Non-contrast CT rapidly excludes hemorrhagic stroke, while CT angiography or MR angiography identifies large vessel occlusions suitable for thrombectomy. Automated tools such as e-ASPECTS have enhanced the accuracy and consistency of early ischemic change detection on non-contrast CT, supporting clinicians in evaluating eligibility for reperfusion therapies in time-critical settings. Advanced imaging techniques, such as CT perfusion or MRI with diffusion and perfusion sequences, assess the ischemic core and penumbra, guiding decisions on extending intervention windows beyond standard time frames in select patients [29,30]. In hemorrhagic stroke, imaging detects hematoma location and size, assesses for mass effect or midline shift, and identifies vascular abnormalities like aneurysms or arteriovenous malformations that may require surgical or endovascular intervention [31].

Imaging also plays a central role in real-time stroke intervention. For instance, intraoperative angiography is used during mechanical thrombectomy to confirm successful recanalization of occluded vessels [32]. Similarly, intraoperative CT or MRI can be employed in neurosurgical procedures to monitor hematoma evacuation or assess complications [33]. Post-treatment, imaging is vital for follow-up care, providing insights into treatment efficacy and detecting complications such as hemorrhagic transformation, edema, or re-occlusion. Long-term imaging follow-ups, such as periodic MR angiography or transcranial Doppler ultrasound, are often employed to monitor for recurrence in patients with conditions like carotid stenosis, aneurysms, or atrial fibrillation. The dynamic integration of imaging into all stages of stroke care underscores its indispensable role in improving patient outcomes [33,34] (Table 1).

## 3. Smart Clothing and Healthcare

Smart clothing, a subset of wearable technology, integrates sensors, electronics, and advanced fabrics to provide continuous and unobtrusive monitoring of physiological and environmental parameters [35]. Unlike traditional wearables such as fitness trackers, smart clothing embeds technology directly into garments, allowing for more comprehensive data collection and comfort. These garments often include conductive fibers, flexible sensors, and microelectronics, enabling real-time tracking of health metrics while maintaining a conventional clothing appearance. Examples include shirts with embedded ECG sensors, socks monitoring foot pressure, and textiles equipped with temperature or hydration sensors [35,36,37].

Smart clothing offers a transformative approach to health monitoring, leveraging its close contact with the body for precise and real-time data collection. These garments can measure metrics such as heart rate, respiratory rate, body temperature, activity levels, and even electrodermal activity to assess stress or hydration levels. Advanced models use stretchable sensors to monitor blood oxygen saturation (SpO2), posture, and muscle activity [37,38]. Wireless connectivity allows data to be transmitted to smartphones or healthcare systems for analysis, enabling remote patient monitoring and early detection of conditions such as arrhythmias, respiratory distress, or dehydration. The seamless integration of smart clothing into daily life positions it as a powerful tool for both preventative healthcare and chronic disease management [38,39].

### 3.1. Smart Clothing Applications for Stroke Patients

Smart clothing equipped with advanced sensors enables early detection and continuous monitoring of health conditions, offering significant advancements in personalized healthcare. These systems are designed to balance precision with patient comfort, ensuring accurate monitoring while being wearable for extended periods. By embedding sensors directly into fabrics, these garments provide non-invasive, real-time data collection on physiological metrics such as heart rate, respiration, temperature, and movement patterns [40]. For example, ECG sensors in smart shirts can detect arrhythmias with high accuracy, while breath-monitoring features identify respiratory distress. Motion-tracking sensors embedded in smart clothing can detect changes in gait or posture, aiding in the early diagnosis of neurodegenerative conditions or stroke-related impairments. The seamless integration into daily wear ensures comfort, making these systems practical for continuous use at home or in clinical settings [40,41,42,43].

However, beyond general benefits, specific applications of smart clothing in stroke patients must be emphasized to reflect their diverse needs across different recovery phases. For acute and subacute stroke patients with motor impairments, garments with embedded EMG (electromyography) sensors—such as BioSignal garments—can track muscle activation during limb movement, allowing therapists to tailor neuromuscular re-education and assess motor function restoration. In patients with gait disorders, Sensoria Smart Socks provide detailed foot pressure mapping, enabling detection of asymmetries or compensatory patterns. This is particularly useful for patients recovering from hemiplegia or drop foot. Post-stroke individuals with high fall risk can benefit from smart belts equipped with accelerometers and gyroscopes that detect balance loss and trigger emergency alerts [44,45,46,47,48,49,50,51,52,53,54].

Cognitively intact but mobility-impaired patients benefit most from wearable ECG-based smart shirts that detect cardiac events like arrhythmias—especially atrial fibrillation, which is associated with recurrent strokes. These garments, such as Hexoskin and Myant Skiin, offer high medical-grade accuracy and are suitable for long-term wear. In contrast, EEG-enabled caps may be more appropriate for patients with cognitive deficits or brainstem strokes where monitoring neural recovery is prioritized. While effective in capturing brainwave anomalies, these devices may be less comfortable for extended daily use. Similarly, smart socks like Sensoria focus on gait asymmetry, useful in post-stroke monitoring or detecting early signs of instability in high-risk individuals. Smart belts, though limited in sensor range, are excellent for fall detection, which may signal neurological impairment [44,45,46,47,48,52,53,54].

When selecting a wearable for early stroke detection, users and clinicians should consider key factors such as sensor accuracy (e.g., ECG and EEG offering medical-grade precision), ease of use (e.g., shirts and socks that can be worn like regular garments), and comfort for long-term daily wear. Battery life, data transmission reliability, and compatibility with AI analytics platforms are also critical for real-time monitoring and intervention. Based on current evidence, ECG-based shirts and gait-monitoring socks present the most practical options for widespread use in stroke risk screening due to their high accuracy, minimal intrusiveness, and user-friendliness. More invasive or specialized options like EEG caps may be better suited for rehabilitation phases rather than early detection. Therefore, an ideal system for early stroke detection would prioritize high-sensitivity cardiac or movement sensors embedded in garments that are easy to don, discreet, and connected to AI-driven platforms capable of detecting early warning signs [6,39,49].

In rehabilitation and recovery tracking, smart clothing offers significant benefits by facilitating remote, detailed monitoring of patient progress. For example, biofeedback provided by motion sensors ensures that exercises are performed correctly, while compliance tracking supports adherence to prescribed rehabilitation programs. These garments demonstrate high sensitivity in detecting subtle movements, enabling therapists to adjust interventions based on precise, real-time data. By bridging the gap between clinical care and home-based management, smart clothing enhances long-term recovery and empowers patients to actively participate in their health journey [40,41,42,43,44,45].

#### 3.1.1. Diagnostics/Detecting Symptoms

In the early detection of stroke symptoms, prompt recognition of warning signs such as sudden numbness, confusion, trouble speaking, vision changes, or severe headache is crucial. Emerging technologies, such as smart clothing embedded with biosensors, can continuously monitor vital signs like heart rate, blood pressure, and mobility patterns, potentially alerting users or healthcare providers to early signs of stroke.

#### 3.1.2. Hexoskin Smart Shirt

The Hexoskin Smart Shirt integrates advanced sensors into a comfortable garment, enabling continuous monitoring of heart rate, respiratory rate, and movement. The accuracy of the sensors is validated for medical applications, offering reliable data on cardiovascular and respiratory functions during stroke recovery [46,47]. Stroke patients, at high risk of cardiovascular events, can benefit from early detection of complications. The shirt’s soft and ergonomic design ensures comfort for long-term wear, making it suitable for both clinical use and daily activities. The ability to provide continuous and unobtrusive health monitoring allows clinicians to adjust rehabilitation strategies proactively, enhancing recovery outcomes [47,48,49,50,51].

#### 3.1.3. Myant Skiin Smart Clothing

Myant Skiin Smart Clothing combines advanced biometric monitoring with everyday functionality [52]. With integrated sensors for tracking ECG, heart rate, and other vitals, these garments provide 24/7 monitoring for stroke patients. The clothing’s comfort and discreet design allow users to wear it throughout daily activities without discomfort, ensuring practical long-term use [53,54]. Continuous cardiac monitoring supports early detection of arrhythmias, reducing the risk of recurrent strokes. The system’s accuracy in detecting irregularities makes it a reliable tool for proactive care [54].

#### 3.1.4. Smart Belts

Smart belts, like those developed by Samsung, detect falls and monitor gait patterns. These belts are lightweight and unobtrusive, ensuring users’ comfort while delivering accurate real-time monitoring [55]. Stroke survivors benefit from immediate alerts during falls, reducing response times and preventing complications. Their precision in detecting movement abnormalities makes them reliable tools for patient safety [55,56].

#### 3.1.5. Smart Textiles for Vital Monitoring

Smart textiles provide continuous monitoring of vital signs like heart rate and respiration through embedded sensors [57]. These textiles are designed to integrate seamlessly into everyday clothing, offering a comfortable and practical solution for long-term health management [57,58,59]. For stroke survivors, the system’s accurate monitoring of physiological metrics supports early detection of potential complications, ensuring timely intervention. The comfort and reliability of these textiles make them ideal for recovery and preventive care [59].

#### 3.1.6. Wearable EEG Caps

Wearable EEG caps monitor brain activity, providing insights into neural recovery during stroke rehabilitation [60]. The lightweight and adjustable design ensures patient comfort, enabling extended use during therapy sessions. EEG caps accurately capture brainwave patterns, helping clinicians assess cognitive and motor recovery. Their portability and precision make them practical for use in both clinical and home settings [61] (Table 2).

### 3.2. Rehabilitation

#### 3.2.1. Sensoria Smart Socks

Sensoria Smart Socks are a breakthrough in mobility monitoring and rehabilitation for individuals with impaired movement. These socks are embedded with textile pressure sensors that offer real-time feedback on foot movement and balance, ensuring accurate gait analysis [62]. The lightweight, comfortable design allows users to wear them throughout daily activities without compromising mobility or convenience. Clinicians can use the detailed gait data to identify walking asymmetries and tailor rehabilitation plans. Stroke survivors benefit from continuous progress tracking, ensuring that therapy remains adaptive and effective [62,63]. The high precision of the sensors ensures reliable data, while the user-friendly design promotes adherence to rehabilitation programs [63].

#### 3.2.2. Rehabilitation Suits (NeuroSuit)

Rehabilitation suits like the NeuroSuit provide mechanical support to enhance muscle control, improve posture, and aid in strength-building exercises. The suits are designed with patient comfort in mind, featuring adjustable, lightweight materials that support natural movement during rehabilitation [64,65]. For stroke patients, these suits enable safe and controlled exercises, promoting neuromuscular re-education and improving mobility. The mechanical precision ensures accurate assistance during movements, reducing the risk of overexertion or strain [66].

#### 3.2.3. BioSignal Garments

BioSignal garments, equipped with electromyography (EMG) sensors, monitor muscle activity in real time, providing valuable insights during rehabilitation [67,68]. These garments are designed for both precision and wearability, ensuring accurate data collection while allowing freedom of movement. Stroke survivors benefit from targeted monitoring of muscle activation, enabling clinicians to tailor therapies effectively. The combination of comfort and high-resolution biofeedback ensures these garments are both practical and clinically impactful [67,68,69].

#### 3.2.4. Smart Gloves for Stroke Rehabilitation

Smart gloves equipped with sensors track finger movements and muscle activity, offering precise feedback during therapy [70,71]. These gloves combine accuracy with an ergonomic design, ensuring ease of use for patients with impaired hand function. Stroke survivors can practice fine motor skills with real-time feedback, fostering better engagement and motivation during recovery. Their high accuracy in monitoring hand movement ensures effective therapy and measurable progress [71,72].

#### 3.2.5. Exoskeleton Suits

Exoskeleton suits assist in regaining mobility by supporting movements such as walking or arm use [73]. The suits are engineered for ergonomic support, with motorized components ensuring accurate assistance tailored to individual needs [73,74]. Stroke survivors benefit from the suits’ ability to promote proper muscle engagement, accelerating recovery. Their combination of functionality and comfort allows for extended use during therapy and daily activities [75,76,77] (Table 3).

## 4. Future of Smart Clothing in Medical Care

Healthcare wearables are undergoing rapid innovation, with smart clothing emerging as a frontier technology that combines comfort, functionality, and precision. Recent trends focus on enhancing sensor miniaturization, flexibility, and durability, allowing for seamless integration of advanced monitoring systems into everyday garments. Innovations include multi-sensor arrays for simultaneous tracking of metrics like heart rate, respiration, hydration, and activity levels, as well as energy-harvesting textiles that eliminate the need for frequent charging [78]. Additionally, the development of washable and stretchable electronic components ensures the durability and usability of smart clothing over extended periods. Emerging applications extend beyond fitness and wellness, targeting areas such as chronic disease management, remote patient monitoring, and early diagnosis of cardiovascular or neurodegenerative conditions [78,79,80].

The integration of artificial intelligence (AI) and data analytics into smart clothing is revolutionizing its potential in healthcare. AI algorithms process the vast amounts of real-time data generated by smart garments, identifying patterns, anomalies, and trends that may indicate early signs of disease or health deterioration [81,82]. For example, machine learning models can analyze ECG data from smart shirts to predict arrhythmias or assess stress levels through electrodermal activity analysis. Predictive analytics enables personalized healthcare interventions, while cloud-based platforms facilitate data sharing with clinicians for enhanced decision-making. AI-powered feedback systems in rehabilitation smart wearables provide real-time corrective guidance to patients, improving the effectiveness of physical therapy. By combining AI with smart clothing, healthcare wearables are evolving into intelligent systems capable of delivering proactive, personalized, and continuous healthcare solutions [81,82,83] (Table 4).

### 4.1. Research and Innovations

Advances in stroke research, medical imaging, and smart wearables are converging to revolutionize diagnosis, treatment, and preventive care. In stroke research, current trends focus on expanding treatment windows through advanced imaging techniques, such as perfusion-based MRI and CT, to identify salvageable brain tissue in ischemic stroke patients. Studies on the efficacy of neuroprotective agents and advanced thrombolytic therapies are also progressing. In imaging, research is prioritizing real-time, portable modalities, such as mobile stroke units equipped with onboard CT scanners, and ultra-fast MRI technologies for emergency stroke diagnosis [25,26]. Simultaneously, smart wearable technologies are being adapted for stroke prevention and recovery, with devices capable of detecting early signs of atrial fibrillation, monitoring blood pressure, and assessing post-stroke rehabilitation progress using motion and activity sensors. These interdisciplinary innovations aim to enhance patient outcomes through early detection and personalized care [65,78,79,80,81].

The future of medical treatment and preventive care lies in integrating advanced technologies like AI, machine learning, and personalized medicine into existing healthcare systems. In stroke care, this includes AI-driven imaging analysis for faster, more accurate diagnosis and individualized treatment plans based on real-time data. Portable, wearable technologies are expected to play a growing role in continuous monitoring, with smart clothing capable of tracking key health metrics and alerting patients and healthcare providers to early warning signs of stroke or cardiovascular events. Preventive care will increasingly emphasize remote monitoring and telemedicine, reducing barriers to access and enabling proactive interventions. Advances in biomaterials may also lead to the development of implantable or even biocompatible sensors that integrate seamlessly into the body. As these technologies converge, the emphasis will shift toward a preventive, patient-centered healthcare paradigm, reducing the burden of diseases like stroke and enhancing overall health outcomes [81,82].

### 4.2. Current Challenges, Potential Solutions

While smart clothing offers promising advancements, its future in medical care faces several challenges that require further exploration. One critical challenge is ensuring the accuracy and reliability of wearable sensors, especially in diverse real-world conditions where motion artifacts and environmental factors can impact data quality [83,84]. Solutions such as advanced signal processing algorithms and sensor fusion techniques could help mitigate these issues. Additionally, scalability and cost-effectiveness remain hurdles for widespread adoption, necessitating innovations in manufacturing processes and materials. Future research could focus on developing low-cost, sustainable, and biodegradable electronic components that maintain functionality without compromising environmental goals [84,85]. Privacy and security of health data collected by smart garments also demand significant attention, as seamless integration with AI and cloud platforms introduces risks of data breaches and unauthorized access. Establishing robust cybersecurity frameworks and implementing decentralized data storage systems like blockchain could address these concerns. Beyond these challenges, future directions should prioritize exploring novel applications, such as integrating smart clothing with advanced therapies like biofeedback and neurostimulation, or enabling its use in underserved areas through solar-powered or energy-efficient designs. By addressing these challenges and expanding research frontiers, smart clothing can realize its full potential as a transformative tool in modern healthcare [83,84,85].

### 4.3. Ethical Considerations in the Use of Smart Clothing for Stroke Care

The integration of smart clothing into stroke care raises several ethical considerations that must be addressed to ensure responsible deployment and equitable outcomes. Data privacy and security represent significant concerns, as these systems collect sensitive health information such as heart rate, movement patterns, and neurological data, which could be vulnerable to breaches or unauthorized access. Ensuring compliance with data protection regulations, such as GDPR or HIPAA, through robust encryption and access control is essential. Furthermore, the accuracy and reliability of smart clothing devices are critical; inaccuracies in sensor readings or algorithmic analyses could lead to misdiagnoses or delayed interventions, potentially harming patients. Rigorous testing and validation protocols are necessary to mitigate this risk. The accessibility and affordability of these technologies also raise ethical questions, as high costs could limit access for economically disadvantaged populations, exacerbating health disparities. Efforts to reduce costs, provide insurance coverage, and ensure inclusivity in device design are essential to address these concerns. Additionally, continuous monitoring through wearable devices may evoke psychological stress or feelings of surveillance among patients, impacting their autonomy and quality of life. To address this, developers must design systems that prioritize user control and minimize unnecessary alerts. Lastly, biases in the algorithms underlying these technologies, particularly those trained on non-representative datasets, could compromise their effectiveness across diverse populations. Including diverse demographic data in algorithm training and performing regular bias audits are necessary to ensure equitable performance. By addressing these ethical considerations, smart clothing can be implemented as a safe, effective, and equitable tool for stroke care [86,87,88,89].

## 5. Discussion

Stroke continues to be a leading cause of disability and mortality worldwide, highlighting the urgent need for precise diagnosis, timely intervention, and effective rehabilitation strategies [1,2,3,4,5,6,7,8,9,10]. Traditional approaches to stroke care, such as the classification of ischemic and hemorrhagic stroke types, provide a foundation for treatment. Advances in medical imaging, including CT and MRI technologies, have significantly improved the ability to diagnose stroke types, assess vascular integrity, and guide interventions like thrombolysis and thrombectomy [18,20]. These innovations emphasize the importance of integrating advanced diagnostics with time-sensitive treatment protocols to optimize patient outcomes [19].

In parallel, emerging wearable technologies, including smart clothing, are poised to transform stroke prevention, rehabilitation, and management. Devices like Sensoria Smart Socks and Hexoskin Smart Shirts enable real-time monitoring of physiological and functional metrics, enhancing the ability to detect anomalies and track recovery [46,47,48]. Importantly, these smart garments generate continuous streams of physiological data—such as ECG, gait patterns, or EEG signals—which can be analyzed using image processing and signal analysis algorithms. These methods, traditionally applied to medical images (e.g., CT or MRI), are increasingly adapted to wearable sensor data to extract meaningful patterns, detect anomalies, and predict stroke-related events. For example, gait heatmaps or postural deviation plots derived from smart socks or belts can be visualized and interpreted using image-based techniques to identify asymmetries or instability indicative of neurological impairment. Thus, image processing serves as a computational bridge that enhances the interpretation of smart clothing outputs for early stroke detection and personalized intervention planning [18,19,20,46,47,48,49,50,51,52,53,54].

The integration into patient care pathways addresses gaps in traditional models by offering continuous, non-invasive monitoring and personalized therapy adjustments. Additionally, the application of smart clothing in rehabilitation, such as NeuroSuit rehabilitation suits and EMG-equipped BioSignal garments, underscores their role in promoting functional recovery and patient engagement [64,65,66]. The psychological impact of continuous physiological monitoring, including potential stress, discomfort, and perceived intrusion, is not thoroughly explored in the current manuscript. These factors are critical, as they may directly affect patient compliance and the sustained use of wearable systems in long-term stroke management. Future studies should systematically investigate these aspects to support the development of patient-centered, acceptable, and effective wearable healthcare solutions.

Wearable technologies are increasingly utilized in stroke detection and monitoring, with systems such as the Hexoskin Smart Shirt and Myant Skiin demonstrating reliable performance in continuous ECG and respiratory tracking, enabling early identification of risk factors like atrial fibrillation. These devices collect physiological data in real time, which is analyzed by integrated AI algorithms to detect anomalies and trigger alerts for timely clinical intervention. In rehabilitation, tools such as Sensoria Smart Socks and EMG-based BioSignal garments monitor gait and muscle activity, providing objective metrics for progress assessment and delivering real-time feedback to correct improper movement execution [46,47,48,49,50,51,52,53,54,55,56,57,58,59,60,61,64,65,66].

These wearable systems are designed with user comfort and ease of use in mind, featuring lightweight, ergonomic materials suitable for daily and long-term wear. Most devices operate wirelessly and require minimal setup, though they typically rely on companion hardware such as smartphones, tablets, or cloud-connected hubs for data transmission. On the software side, dedicated mobile applications and AI-based platforms are essential for data visualization, anomaly detection, and integration with electronic health records to support clinical decision-making [86,87,89].

The future of stroke care is likely to be shaped by the convergence of advanced imaging modalities, wearable technologies, and artificial intelligence (AI). AI-driven analytics can harness data from wearables and imaging to provide predictive insights and tailor interventions to individual patients, marking a shift toward proactive, personalized care [79,80]. However, challenges remain, including the need for cost-effective solutions, data security, and integration into existing healthcare systems. A more structured understanding of sensor accuracy, long-term wearability, data privacy concerns, and real-world integration challenges emerged across the reviewed studies, suggesting that while the potential is strong, implementation requires multidisciplinary coordination. Continued interdisciplinary research and innovation are essential to bridge these gaps, paving the way for a more effective, patient-centered approach to stroke management [83].

## 6. Limitations

The integration of smart clothing and advanced medical imaging technologies into stroke care faces significant challenges. High implementation costs and the need for specialized infrastructure can limit accessibility, particularly in resource-constrained settings, raising concerns about equitable adoption. Data privacy and security risks associated with continuous health monitoring necessitate robust regulatory compliance and cybersecurity measures. Technological limitations, including sensor accuracy, durability, and the need for recalibration, may hinder reliability and clinical acceptance. Additionally, integrating wearable device data into existing healthcare workflows requires updates to electronic health record systems and clinician training, adding to logistical complexity. Patient adherence is influenced by usability, comfort, and social perceptions, potentially limiting the effectiveness of wearable devices, especially for older adults or those with severe impairments. The lack of large-scale clinical validation and standardized protocols further complicates their adoption in routine practice. Evolving regulatory frameworks and ethical concerns, such as continuous monitoring and predictive analytics, require careful consideration to align with clinical standards and patient expectations. Addressing these barriers will require multidisciplinary collaboration to enhance affordability, usability, and interoperability while ensuring robust data governance. Such efforts are crucial for realizing the potential of these technologies in transforming stroke care.

## 7. Conclusions

Stroke remains a critical global health challenge, necessitating timely diagnosis, effective treatment, and comprehensive rehabilitation strategies. Advances in medical imaging have significantly enhanced stroke care by enabling rapid differentiation of ischemic and hemorrhagic types, precise localization of brain injury, and informed therapeutic decision-making. In parallel, wearable technologies—particularly smart clothing—are showing real promise for use across all stages of stroke care, from early risk detection to long-term recovery monitoring.

Devices such as Sensoria Smart Socks, Hexoskin Smart Shirts, Myant Skiin garments, and EEG-integrated caps are not only technically feasible but also clinically relevant for real-time physiological monitoring. These tools offer the possibility of continuous, non-invasive tracking of key metrics such as heart rate, gait, respiration, and brain activity, supporting personalized rehabilitation and early detection of complications. Smart clothing can complement traditional imaging and clinical assessments, especially by enabling remote patient monitoring and extending care beyond hospital settings. Although large-scale clinical validation is still limited, current evidence supports their potential for integration into stroke monitoring protocols, particularly in outpatient and home care scenarios.

To realize this potential, future research should focus on developing cost-effective, durable, and user-friendly smart garments that integrate seamlessly with clinical-grade systems and electronic health records. Large-scale clinical trials are essential to establish efficacy, define standardized use protocols, and assess their impact on functional outcomes. Furthermore, challenges related to data privacy, long-term wearability, and patient adherence must be addressed to ensure these technologies are both effective and ethically sound. If these barriers are overcome, smart clothing could become a valuable and practical addition to stroke care, offering a scalable solution for continuous monitoring and individualized support.

## Figures and Tables

**Table 1:** Comprehensive Overview of Stroke Classification, Symptoms, Diagnosis, Imaging Modalities, and Treatment Analysis—Key Points for Effective Stroke Management and Patient Outcomes.

Section	Key Points
Overview of Stroke Types	− Stroke is caused by disrupted blood flow to the brain, leading to neurological damage. − Ischemic Stroke (87% of cases): Caused by obstruction of cerebral blood flow (e.g., thromboembolism, atherosclerosis). − Hemorrhagic Stroke (13% of cases): Caused by ruptured blood vessels (e.g., hypertension, aneurysmal rupture). − Risk factors: Atrial fibrillation, hypertension, diabetes, smoking, sedentary lifestyle, genetic predisposition.
Stroke Symptoms and Diagnosis	− Symptoms: Sudden weakness/numbness (one side), aphasia, facial drooping, vision changes, severe headache (hemorrhagic stroke), loss of balance. − Public awareness: FAST (Face drooping, Arm weakness, Speech difficulties, Time to call emergency services). − Diagnosis: Non-contrast CT (first-line), MRI (sensitive to early ischemic changes), CT/MR angiography, transcranial Doppler, cardiac evaluations.
Imaging Modalities for Diagnosis	− Non-contrast CT: Differentiates ischemic (hypoattenuation) from hemorrhagic stroke (hyperdense regions). − CT Perfusion/Angiography: Assesses blood flow, vascular integrity, and salvageable tissue. − MRI: Diffusion-weighted imaging for ischemia, FLAIR for older infarcts, MR angiography for vasculature. − Complementary techniques: Cerebral angiography, transcranial Doppler ultrasound.
Analysis of Imaging for Treatment	− Guides eligibility for interventions: − Ischemic Stroke: Thrombolysis (tPA), thrombectomy (large vessel occlusions). − Hemorrhagic Stroke: Hematoma assessment, vascular abnormalities (e.g., aneurysms). − Real-time intervention: Intraoperative angiography for thrombectomy, CT/MRI during surgery. − Post-treatment imaging: Monitors efficacy, complications (e.g., re-occlusion, hemorrhagic transformation).

**Table 2:** Comprehensive evaluation of advanced wearable technologies in stroke management for diagnostics and detecting—advantages, disadvantages, sensory accuracy, ergonomic design, and long-term wearability.

Aspect	Advantages	Disadvantages	Sensor Accuracy	Ergonomic Design	Long-Term Wearability
Hexoskin Smart Shirt	Continuous monitoring of heart rate, respiratory rate, and movement; validated for medical accuracy; enhances proactive rehabilitation strategies.	Relies on consistent wear for effective monitoring; may not provide detailed muscle activity data.	High accuracy validated for medical applications.	Soft, comfortable design for long-term wear.	Designed for long-term use during daily activities and clinical settings.
Myant Skiin Smart Clothing	24/7 monitoring of ECG and vitals; discreet and comfortable for daily wear; supports early detection of arrhythmias.	Limited to biometric and cardiac monitoring; may lack gait or movement analysis capabilities.	Reliable accuracy for ECG and cardiac monitoring.	Discreet and practical; ensures user comfort.	Comfortable for everyday wear; suitable for prolonged monitoring.
Smart Belts	Real-time fall detection; lightweight and unobtrusive; immediate alerts reduce response time during emergencies.	Primarily focused on fall detection; less comprehensive for other health parameters.	Precise in fall and gait abnormality detection.	Lightweight and unobtrusive design.	Highly wearable due to its lightweight nature; suitable for continuous use.
Smart Textiles for Vital Monitoring	Seamless integration into everyday clothing; continuous monitoring of vitals like heart rate and respiration; supports early detection of complications.	Potential limitations in advanced rehabilitation applications; requires proper maintenance for long-term use.	High accuracy in monitoring physiological metrics.	Seamlessly integrates into everyday clothing.	Comfortable and practical for long-term health management.
Wearable EEG Caps	Accurately captures brainwave patterns; portable and suitable for both clinical and home settings; enables insights into neural recovery.	May feel intrusive for prolonged use; primarily focused on neural activity rather than holistic health metrics.	Highly precise in detecting brainwave patterns.	Lightweight and adjustable for user comfort.	Suitable for extended therapy sessions; may need adjustments for comfort.

**Table 3:** Comprehensive evaluation of advanced wearable technologies in stroke management for rehabilitation—advantages, disadvantages, sensory accuracy, ergonomic design, and long-term wearability.

Aspect	Advantages	Disadvantages	Sensor Accuracy	Ergonomic Design	Long-Term Wearability
Sensoria Smart Socks	Provides real-time feedback on foot movement and balance; accurate gait analysis; promotes adherence to rehabilitation programs.	Limited to foot movement and balance monitoring; may not address upper-body rehabilitation needs.	High precision in gait and foot movement analysis.	Lightweight, comfortable design for daily wear.	Comfortable for extended use during daily activities and rehabilitation.
Rehabilitation Suits (NeuroSuit)	Enhances muscle control, posture, and strength; allows safe, controlled exercises; reduces risk of overexertion or strain.	Requires proper fit and adjustment; may be costly and bulky for some users.	Mechanically precise in assisting and monitoring movements.	Adjustable, lightweight materials for patient comfort and natural movement.	Suitable for repeated use during therapy sessions; comfort depends on proper adjustment.
BioSignal Garments	Monitors muscle activity in real time; enables precise and targeted therapy; offers high-resolution biofeedback.	Primarily focused on muscle activity; limited for overall cardiovascular or neural monitoring.	Highly accurate in monitoring muscle activity with EMG sensors.	Designed for precision and wearability, allowing freedom of movement.	Practical for long-term rehabilitation due to its wearable and flexible design.
Smart Gloves for Stroke Rehabilitation	Tracks finger movements and muscle activity; fosters better engagement and measurable progress in fine motor skill recovery.	Limited to hand and finger rehabilitation; may not address broader mobility issues.	High accuracy in tracking hand and finger movements.	Ergonomic and easy to use for patients with impaired hand function.	Ensures ease of use and comfort for long-term hand therapy.
Exoskeleton Suits	Supports movements like walking and arm use; promotes proper muscle engagement; accelerates recovery effectively.	May require extensive setup and calibration; potentially expensive and less accessible for home use.	Highly accurate in supporting and monitoring full-body movements.	Engineered for ergonomic support and motorized assistance.	Designed for extended use during therapy and daily activities, though setup may limit convenience

**Table 4:** Comparison of image-based analysis and smart clothing technologies in stroke diagnosis and treatment.

Aspect	Image-Based Analysis (CT, MRI, etc.)	Smart Clothing Technologies
Primary Use	Acute diagnosis, localization of stroke, eligibility assessment for treatments like thrombolysis or thrombectomy	Continuous real-time monitoring, early symptom detection, rehabilitation tracking
Technology Types	CT, MRI, angiography, Doppler ultrasound, perfusion imaging	Smart shirts (e.g., Hexoskin), EEG caps, smart socks, biosignal garments, exoskeletons
Time Sensitivity	High—rapid imaging required in acute stroke cases (“time is brain”)	Useful for long-term monitoring and early detection, but not for immediate acute diagnosis
Data Output	High-resolution anatomical and functional images; spatially accurate	Continuous physiological data: heart rate, gait, brainwaves, respiration, muscle activity
Clinical Setting	Requires hospital-based imaging infrastructure	Can be used at home, during daily activities, or in rehab settings
AI Integration	Supports image interpretation (e.g., e-ASPECTS) and triage	AI enables anomaly detection, rehabilitation feedback, predictive analytics
Patient Involvement	Passive—requires patient transport and technician operation	Active—worn continuously, patient engagement in rehabilitation encouraged
Strengths	Precise diagnosis; guides emergency interventions; visual confirmation of stroke type and location	Enables proactive care; supports long-term monitoring and therapy adjustments; enhances accessibility and continuity of care
Limitations	Limited portability; exposure to radiation (CT); requires clinical expertise	May have variable sensor accuracy; requires adherence; limited in detecting anatomical changes directly
Ideal Phase of Care	Acute diagnosis and treatment planning	Prevention, rehabilitation, remote follow-up
